# Phytoplankton Diversity, Spatial Patterns, and Photosynthetic Characteristics Under Environmental Gradients and Anthropogenic Influence in the Pearl River Estuary

**DOI:** 10.3390/biology13070550

**Published:** 2024-07-22

**Authors:** Jing Xia, Haojie Hu, Xiu Gao, Jinjun Kan, Yonghui Gao, Ji Li

**Affiliations:** 1 School of Oceanography, Shanghai Jiao Tong University, Shanghai 200030, China; xiajingsherry@sjtu.edu.cn (J.X.); huhaojie@sjtu.edu.cn (H.H.); shayegao@sjtu.edu.cn (X.G.); 2 Stroud Water Research Center, 970 Spencer Rd., Avondale, PA 19311, USA; jkan@stroudcenter.org; 3 Key Laboratory of Polar Ecosystem and Climate Change, Ministry of Education, School of Oceanography, Shanghai Jiao Tong University, Shanghai 200030, China; 4 Key Laboratory for Polar Science, Polar Research Institute of China, Ministry of Natural Resources, Shanghai 200136, China

**Keywords:** estuary, phytoplankton, 18S rRNA, environmental factors, photosynthesis

## Abstract

**Simple Summary:**

The Pearl River Estuary (PRE) in China, a highly urbanized coastal area, presents a unique opportunity to study the effects of environmental changes on the phytoplankton com-munity. In September 2018, a field study was conducted to examine how the photosynthetic status and spatial distribution of these organisms varied from freshwater to oceanic waters. Dinophyta and Haptophyta were prevalent in seawater, while Chlorophyta and Cryptophyta dominated from freshwater to estuarine water. Bacillariophyta were found across all regions. Phytoplankton in the mixing zone showed signs of stress due to fluctuating environmental conditions, whereas those in freshwater and oceanic areas appeared more photosynthetically active. Human activities have in-creased nutrient levels in the estuary, leading to higher chlorophyll concentrations and more diverse phytoplankton communities upstream. Understanding these patterns helps us assess the health of coastal ecosystems, which is crucial for managing the impacts of climate change and human development on marine environments.

**Abstract:**

The Pearl River Estuary (PRE) is one of the world’s most urbanized subtropical coastal systems. It presents a typical environmental gradient suitable for studying estuarine phytoplankton communities’ dynamics and photosynthetic physiology. In September 2018, the maximum photochemical quantum yield (*Fv*/*Fm*) of phytoplankton in different salinity habitats of PRE (oceanic, estuarine, and freshwater zones) was studied, revealing a complex correlation with the environment. *Fv*/*Fm* of phytoplankton ranged from 0.16 to 0.45, with taxa in the upper Lingdingyang found to be more stressed. Community composition and structure were analyzed using 18S rRNA, accompanied by a pigment analysis utilized as a supplementary method. Nonmetric multidimensional scaling analysis indicated differences in the phytoplankton spatial distribution along the estuarine gradients. Specificity-occupancy plots identified different specialist taxa for each salinity habitat. Dinophyta and Haptophyta were the predominant taxa in oceanic areas, while Chlorophyta and Cryptophyta dominated freshwater. Bacillariophyta prevailed across all salinity gradients. Canonical correlation analysis and Mantel tests revealed that temperature, salinity, and elevated nutrient levels (i.e., NO_3_^−^-N, PO_4_^3−^-P, and SiO_3_^2−^-Si) associated with anthropogenic activities significantly influenced the heterogeneity of community structure. The spatial distribution of phytoplankton, along with in situ photosynthetic characteristics, serves as a foundational basis to access estuarine primary productivity, as well as community function and ecosystem health.

## 1. Introduction

Estuaries serve as transitional zones connecting freshwater and marine environments [1,2,3,4], where complex hydrodynamics create various physical and chemical habitats, thus shaping the diversity of freshwater and saltwater organisms. A typical feature of estuaries is the salinity gradient formed by the mixing of two distinct water masses, which strongly influences community composition and diversity [1,2,3,5,6,7]. The physiochemical properties in estuaries, such as light availability, salinity, and nutrient availability, dynamically regulate the photosynthetic carbon fixation, biomass, and community composition of phytoplankton, resulting in spatial–temporal heterogeneity in phytoplankton distribution [5,8,9,10,11]. In addition, site-specific disturbance, climatology, and nutrient enrichment account for large-scale patterns of phytoplankton variability [12]. In recent decades, as estuaries accumulate both natural and excessive anthropogenic inputs of nutrients, there has been a trend of eutrophication, leading to an increase in phytoplankton biomass and the occurrence of harmful algal blooms [13,14]. As the primary producers in aquatic ecosystems, phytoplankton play a pivotal role not only in shaping the food webs but also in regulating the biogeochemical cycles. Indeed, a comprehensive understanding of the relationship between environmental conditions and phytoplankton composition is critical for the carbon cycles in the estuaries [15].

Phytoplankton photosynthesis accounts for most aquatic primary production, regulating phytoplankton growth, population dynamics, community composition, and succession [16,17]. Estuarine environmental gradients affect phytoplankton photosynthesis and metabolism, leading to variations in carbon assimilation and primary production [10,18,19,20]. Active variable chlorophyll fluorescence is a highly sensitive and rapid measurement of phytoplankton photosynthetic activity, indicating the impact of environmental stress, such as nutrient limitation and light inhibition [19,21,22]. The maximal photochemical efficiency (*Fv*/*Fm*) is a well-recognized indicator for assessing photosynthetic performance, directly reflecting the efficiency of photons that are converted into electrons by phytoplankton [16,19,23]. The *Fv*/*Fm* ratios typically show substantial variability across different marine environments, with increases from stressful to favorable conditions [16,17,19,24]. Specifically, *Fv*/*Fm* values can reach up to 0.65 in nutrient-rich areas and decrease below 0.3 in oligotrophic regions [24,25]. Overall, chlorophyll fluorescence contributes significantly to aquatic ecosystem studies, providing significant insights into the photochemical mechanisms of phytoplankton [10,16,17].

The Pearl River is China’s third longest river with the second largest discharge, forming complex branches over its watershed and entering the South China Sea through eight outlets [26,27]. The Pearl River Estuary (PRE) is influenced by both the river discharge and the South China coastal current, contributing to its complex estuarine dynamics [26]. The Pearl River Delta urban agglomeration is one of the three major city clusters in China, with a high concentration of industries and an urbanization rate of 85.3% in 2018, resulting in a significant increase in urban land use [28,29]. Over the past 20 years, its population has increased by 1.8 times, reaching 78.01 million in 2020 [30]. These changes have contributed to the large nutrient loading into the PRE [31,32]. The average dissolved inorganic nitrogen (DIN) increased from 2.05 × 10^−5^ t/a in 1985–1995 to 3.91 × 10^−5^ t/a in 2014–2021 [32]. Dissolved inorganic phosphate (DIP) has also gradually increased over the past 20 years [31]. Consequently, eutrophication has caused frequent algal blooms [33]. The hydrology, physical transport, and biogeochemical processes of this region have been extensively investigated [27,34,35], along with records and descriptions of the spatial and temporal distribution of phytoplankton [7,8,36]. However, there is a lack of studies on the spatial patterns of the photosynthetic physiology of phytoplankton in this estuary and its connections with the environment and community. Understanding the spatial patterns of phytoplankton community structure in the PRE, along with the distribution characteristics of photosynthetic physiology related to primary productivity under environmental gradients and human impacts, is crucial for comprehending the responses of estuarine and coastal ecosystems to global climate change. The integration of active chlorophyll fluorescence with multiple analytical methods can facilitate a comprehensive understanding of phytoplankton physiology and community composition [37,38,39]. The application of 18S ribosomal RNA genes analysis provides a novel approach for gaining insights into the composition of eukaryotic unicellular phytoplankton communities [39,40]. Additionally, chemotaxonomy based on photosynthetic pigments is often used to characterize phytoplankton biomass and taxonomic groups [41,42,43]. This study employs high-throughput sequencing complemented by photosynthetic pigment analysis to access the spatial patterns of phytoplankton in the PRE. Concurrently, *Fv*/*Fm* is used as an indicator of the photosynthetic characteristics, providing a comprehensive investigation of the health status of the phytoplankton community under natural and anthropogenic stressors. This study aims to investigate the changes in the population structure and function of primary producers in a typical subtropical eutrophic estuary, providing baseline data for the response of local or similar highly urbanized estuarine ecosystems to climate change and offering factual evidence for coastal management policies.

## 2. Methods and Materials

### 2.1. Study Sites and Sample Collection

**Figure 1:**
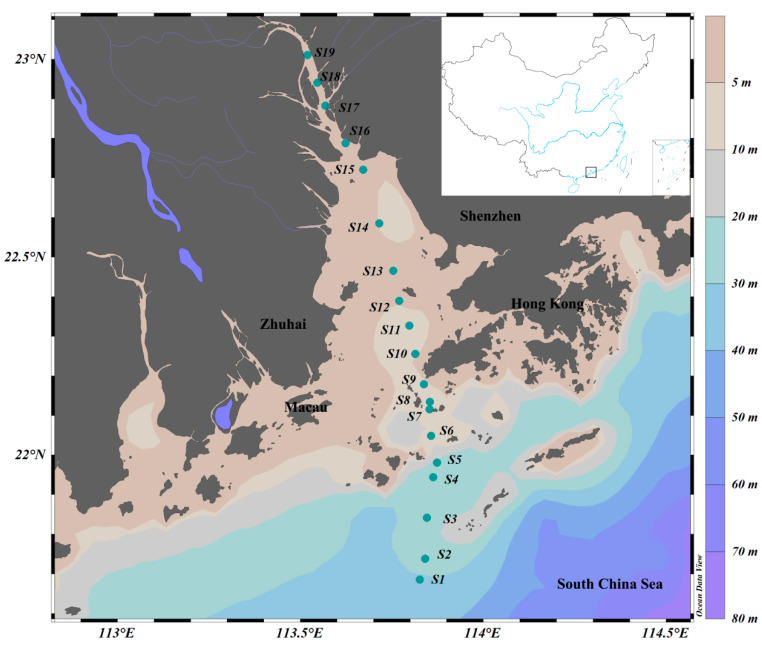
Sampling stations in the Pearl River Estuary. Color gradients represent ocean bathymetry.

A comprehensive biological survey was conducted in the PRE on R.V. Haiyang-Dizhi 10 during 27–28 September 2018 (Figure 1). Water samples were collected using an underway system that consisted of a pump mounted at a depth of approximately 4 m. Based on phytoplankton biomass, 150–250 mL of water samples was filtered through each GF/F filter (25 mm, Whatman, UK) using a low-pressure vacuum pump. At least 3 filter samples were collected at each station, and the filters were stored in sterile bags and preserved at −80 °C in the dark until subsequent high-performance liquid chromatography (HPLC) and high-throughput sequencing. The filtrate water was stored in clean polyethylene bottles at −20 °C for nutrient analysis.

### 2.2. Determination of Environmental Parameters

The water properties of temperature and salinity were measured using a thermosalinograph (SBE45, Sea-Bird Inc., Bellevue, WA, USA). Nitrate (NO^3−^-N) and silicate (SiO_3_^2−^-Si) were determined using a SEAL Analytical AQ400 autoanalyzer (Seal Analytical, Mequon, WI, USA). Ammonium (NH_4_^+^-N) was measured by hypobromite oxidation spectrophotometry at 540 nm, and phosphate (PO_4_^3−^-P) was determined by molybdenum blue spectrophotometry at 882 nm [44]. The composition of photosynthetic pigments was analyzed by high-performance liquid chromatography (Dionex UltiMate 3000, Thermo Fisher Scientific, Waltham, MA, USA), according to the method of Van Heukelem and Thomas [45].

Stations were divided according to salinity: oceanic water (salinity ≥ 30; S1~S4), estuarine water (salinity 5~30; S5~S13 in the Lingdingyang area), and freshwater (salinity < 5; S14~S19).

### 2.3. Measurement of In Situ Photosynthetic Activity

Water samples were stored in brown bottles immediately after collection for 15 min dark adaption. Subsequently, the maximum photochemical quantum yield (*Fv*/*Fm*) of photosystem II (PS II) was measured using a Phyto-PAM II (Heinz Walz, Effeltrich, Germany). The minimal fluorescence level (*F*_0_) was first measured under minimum light (~0 µmol/(s·m^2^), and then a saturating pulse (SP) was applied to obtain the maximal fluorescence level (*Fm*). The *Fv*/*Fm* ratio was calculated using Equation (1) [46]:

(1) $$F\nu /Fm=\frac{Fm-F_{0}}{Fm}$$

### 2.4. DNA Extraction, PCR Amplification, and Sequencing

The collected filters were used for high-throughput sequencing. Total genomic DNA was extracted using the CTAB method according to the manufacturer’s instructions, and stored at −20 °C for further analysis. The quality and concentration of the extracted DNA were assessed using a NanoDrop NC2000 spectrophotometer (Thermo Fisher Scientific, Waltham, MA, USA) and agarose gel electrophoresis. Polymerase chain reaction (PCR) was subsequently performed on the DNA samples.

The 18S rRNA genes V4 region was amplified through polymerase chain reaction (PCR) with forward primer 547F (5′-CCAGCASCYGCGGTAATTCC-3′) and reverse primer V4R (5′-ACTTTCGTTCTTGATYRA-3′) [47]. The 25 µL PCR components contained 5 µL of buffer (5×), 0.25 µL of FastPfu DNA Polymerase (5U/µL), 2 µL (2.5 mM) of dNTPs, 1 µL (10 µM) of each forward and reverse primer, 1 µL of DNA template, and 14.75 µL of ddH_2_O. The PCR amplification protocol featured an initial denaturation at 98 °C for 3 min, followed by 33 cycles at 98 °C for 30 s, 46 °C for 30 s, and 72 °C for 45 s, with a final extension at 72 °C for 5 min. PCR amplicons were purified with Vazyme VAHTSTM DNA Clean Beads (Vazyme, Nanjing, China) and quantified using the Quant-iT PicoGreen dsDNA Assay Kit (Invitrogen, Carlsbad, CA, USA). Amplicons were pooled in equal amounts. DNA samples were processed by Shanghai Personal Biotechnology Co., Ltd. (Shanghai, China), and paired-end 2 × 250 bp sequencing was performed using the Illlumina NovaSeq platform (San Diego, CA, USA) with the NovaSeq 6000 SP Reagent Kit (500 cycles).

### 2.5. Sequencing Data Processing

The obtained 18S rRNA sequencing data were processed using Quantitative Insights Into Microbial Ecology (QIIME2, version 2019.4) [48]. The demux plugin was utilized for demultiplexing the sequencing libraries, and the cutadapt plugin was employed to remove primers [49]. Sequence quality filtering, denoising, merging, and chimera removal were conducted using DADA2 [50]. The resulting Amplicon Sequence Variants (ASVs) table had singleton ASVs removed. Taxonomic classification of the ASVs was performed against the National Center for Biotechnology Information (NCBI) database (https://www.ncbi.nlm.nih.gov/, accessed in August 2019) using the classify-sklearn Naive Bayes taxonomy classifier within the feature-classifier plugin [51]. ASVs represented by protozoa, fungi, and macroalgae were removed before rarefaction. The raw sequencing data were uploaded to NCBI, with the accession number PRJNA1101256.

### 2.6. Statistical Analysis and Visualization

MATLAB (2023b, Natick, MA, USA) and Ocean Data View (version 5.6.7, https://odv.awi.de, accessed on 25 October 2023) [52] were used for map visualization. Statistics and visualization were performed in R Studio (version 4.2.2). The “rstatix” and “FSA” packages were used to perform Kruskal–Wallis tests and Dunn’s multiple comparisons test on environmental factors.

Alpha diversity indices (Chao1, Simpson, Shannon, Pielou’s evenness, Faith’s PD, and observed species richness) were calculated using GenesCloud tools (https://ww w.genescloud.cn, accessed on 28 February 2024) and subjected to Kruskal–Wallis tests and Dunn’s multiple comparisons test. The “vegan” package (version 2.6-4) was employed to plot rarefaction curves to assess sequencing saturation. The relative abundances at the phylum and genus levels of phytoplankton were calculated using the “plyr” and “reshape2” packages, and visualized using the “ggplot2” and “circlize” packages [53]. Differences in phytoplankton community structure among salinity habitats were evaluated using non-metric multidimensional scaling (NMDS) based on Bray–Curtis distance with the environmental fitting test analysis (envfit) method, followed by an analysis of similarities (ANOSIM). Specificity–occupancy (SPEC–OCCU) plots were drawn following the method described by Gweon et al. [54] to identify potential key species of phytoplankton. To compare the correlation between environmental factors, major phytoplankton phyla, and photosynthetic physiological parameters along the salinity gradient, the “psych” package was used for Spearman correlation analysis. To evaluate the relationship between overall community structure and environmental factors, detrended correspondence analysis (DCA) was performed on species data transformed via Hellinger transformation, and canonical correlation analysis (CCA) was employed due to the length of the first axis being greater than 4. During CCA analysis, the explained variance was calculated after correcting for R^2^, and all constrained axes were tested for significance using permutation tests. After that, Monte Carlo permutation tests were used to assess the significance of environmental factors on community impact. Mantel tests were performed using the “ggcor” package.

CHEMTAX [55] was used to calculate the relative abundances of phytoplankton at the phylum level based on HPLC analysis, using a matrix adapted from Wang, et al. [53] in their study of the South China Sea in R studio.

## 3. Results

### 3.1. Physical and Chemical Property Along the PRE

**Figure 2:**
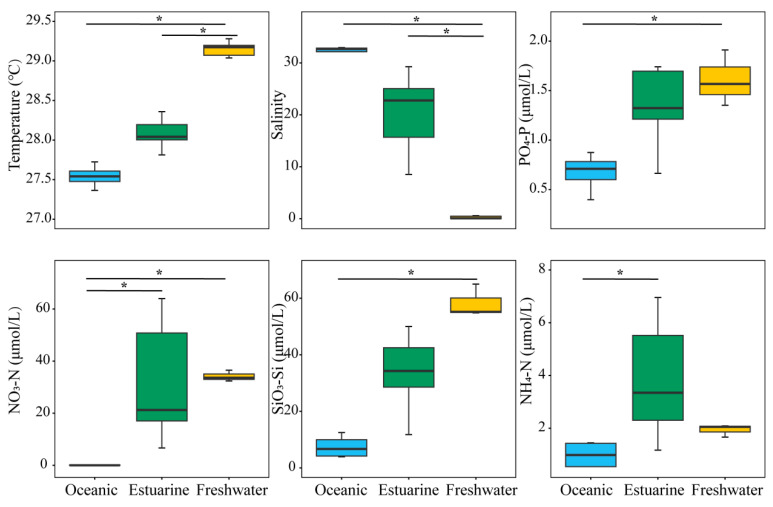
Variation in environmental factors across different regions of the Pearl River Estuary based on salinity levels. “*” denotes statistically significant differences at *p* < 0.05.

Investigations on environmental factors were conducted along the PRE (Figure 2). Results revealed significant differences (*p* < 0.05) in the distributions of temperature, salinity, and nutrients from the lower to the upper estuary, displaying a distinct gradient trend (Figure 2). Concentrations of PO_4_^3−^-P and SiO_3_^2−^-Si, associated with temperatures, decreased significantly from the upper estuary to the lower estuary (*p* < 0.05). Significant differences in environmental factors, except for NH_4_^+^-N, were observed between oceanic and freshwater zones (*p* < 0.05). NO_3_^−^-N and NH_4_^+^-N concentrations were generally higher in the upper estuary, and the nitrogen levels in estuarine water were significantly higher than those in oceanic water (*p* < 0.05).

### 3.2. Distribution of Phytoplankton Photosynthetic Parameters

**Figure 3:**
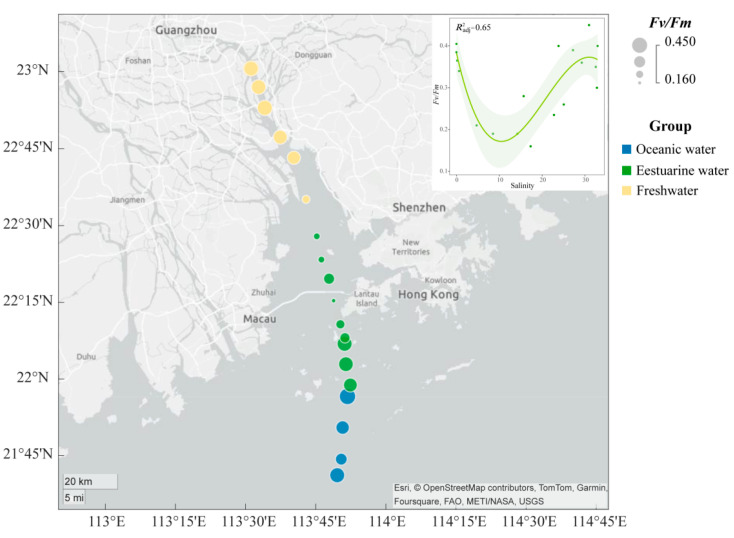
Variation in maximum photochemical quantum yield (*Fv*/*Fm*) of phytoplankton in the Pearl River Estuary. A smooth-fit curve showing *Fv*/*Fm* as a function of salinity.

The spatial distribution of the *Fv*/*Fm* of the phytoplankton community in the PRE (Figure 3) exhibits a “U-shaped” trend along the salinity gradient. The *Fv*/*Fm* values ranged from 0.16 to 0.45, with higher photosynthetic activity in the oceanic zone (average 0.38) and the upper estuary (average 0.35) compared to the tidal mixing water of the lower estuary (average 0.27) (Figure 3).

### 3.3. Spatial Variation in the Taxonomic Composition of the Phytoplankton Community

**Figure 4:**
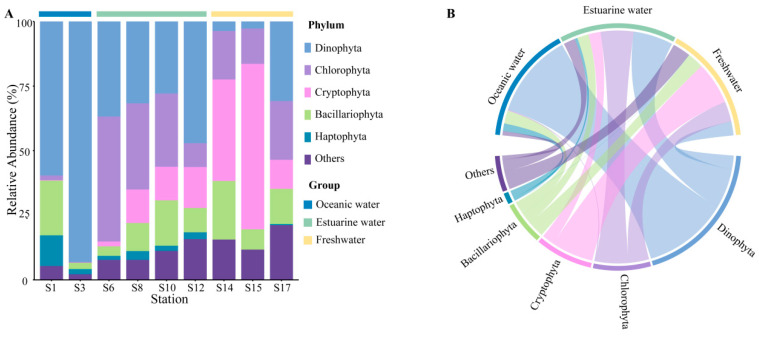
Phytoplankton community composition at the phylum level in the Pearl River Estuary based on 18S rDNA sequencing. (**A**) Relative abundance. (**B**) Chord diagram illustrating species–sample relationships.

Rarefaction curves for different samples (Figure S1) showed a plateau at a reads number of 20,000, indicating that the sequencing depth was representative of the majority of species. The 18S rRNA sequencing results exhibited the composition of phytoplankton at the phylum level, which varied regionally across the PRE (Figure 4), including Dinophyta (average relative abundance 37.06%), Chlorophyta (19.61%), Cryptophyta (17.60%), Bacillariophyta (12.14%), and Haptophyta (2.65%). Dinophyta (an average relative abundance of 76.36%) was the predominant group in oceanic areas (Figure 4B), followed by Bacillariophyta (11.81%) and Haptophyta (6.92%). The abundance of Dinophyta decreased with reducing salinity, from 35.88% in estuarine areas to 12.42% in freshwater areas. Similarly, Haptophyta followed this trend (Figure 4). Conversely, the relative abundance of Cryptophyta and Chlorophyta significantly increased in the upper estuary, (Figure 4), becoming the dominant group in estuarine and freshwater areas. The relative abundance of Bacillariophyta showed minor variations across different salinity habitats.

At the genus level, the representative taxonomy of each salinity habitat exhibited significant differences (Table S1). *Gyrodinium*, *Chaetoceros*, and *Chrysochromulina* are the primary representative groups in oceanic areas. *Chlorella*, *Cyclotella*, and *Ostreococcus* are abundant in mixed areas. In freshwater areas, *Cryptomonas*, *Cyclotella*, and *Chlamydomonas* were the dominant species, with *Cryptomonas* particularly accounting for 25.81%.

**Figure 5:**
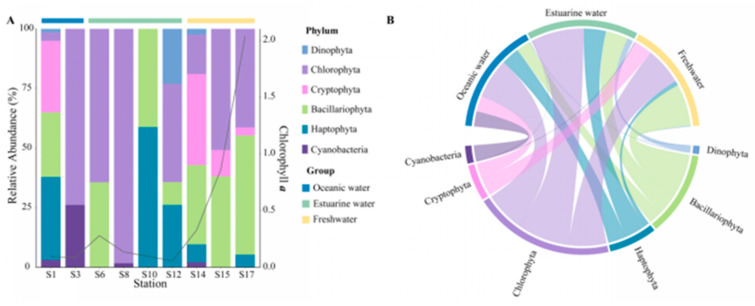
Phytoplankton community composition at the phylum level in the Pearl River Estuary based on pigment analysis. (**A**) Relative abundance. (**B**) Chord diagram illustrating species–sample relationships.

The community composition, derived from pigment analysis, in the PRE (Figure 5), showed that Cyanobacteria were only present at stations S1, S3, S8, and S14 (Figure 5A), with higher abundances noted in high salinity areas (Figure 5B). Both methods showed that Dinophyta and Haptophyta predominantly inhabited polyhaline and euhaline zones, and Bacillariophyta were widely distributed along the salinity gradient, while Cryptophyta tended to inhabit mesohaline and oligohaline zones.

### 3.4. Spatial Patterns of Phytoplankton Community Diversity

Table 1 displays the alpha diversity indices of different salinity habitats, including Chao1, Simpson, Shannon, Pielou’s evenness, Faith’s PD, and observed species richness. The freshwater-oligohaline zone had the highest average values for all diversity indices, without significant differences among the three habitats (*p* > 0.05).

**Figure 6:**
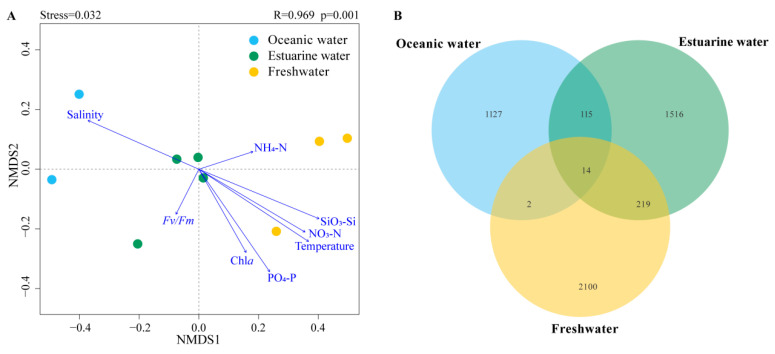
(**A**) NMDS (Non-metric multidimensional scaling) with envfit method of phytoplankton communities across different salinities; R and *p* values were calculated by an analysis of similarities (ANOSIM). (**B**) Venn diagram illustrating phytoplankton distribution across different salinity levels.

NMDS based on Bray–Curtis distance revealed that the stress value was less than 0.05, and samples from different salinity habitats clustered well together, clearly separating into three distinct clusters with good interpretability (Figure 6A). Results from ANOSIM also indicated significant differences in phytoplankton community composition between oceanic, estuarine, and freshwater zones, with clear distinctions (*p* < 0.05) (Figure 6A). To reveal the environmental gradient and the relationship between the photosynthetic physiological activity of phytoplankton (*Fv*/*Fm)* and community structure, the envfit method was performed (Figure 6A). Nutrient levels were negatively correlated with salinity, which significantly affected phytoplankton communities in freshwater areas. *Fv*/*Fm* was negatively correlated with the estuarine population, indicating that the photosynthetic activity of the phytoplankton community in the mixed zone was lower, which may be related to phytoplankton cellular stress caused by the drastic change in salinity.

Venn diagrams showing the overlap of phytoplankton taxonomic groups among different salinity habitats are presented in Figure 6B. The freshwater zone had the highest number of ASVs, totaling 2335, while the oceanic zone had the fewest. There were 14 ASVs common across the three salinity zones. The freshwater and estuarine zones shared more ASVs, totaling 233; however, the overlap with the oceanic zone was the least, with only 16 shared ASVs.

### 3.5. Differences in Phytoplankton Community Composition across Different Salinity Habitats

**Figure 7:**
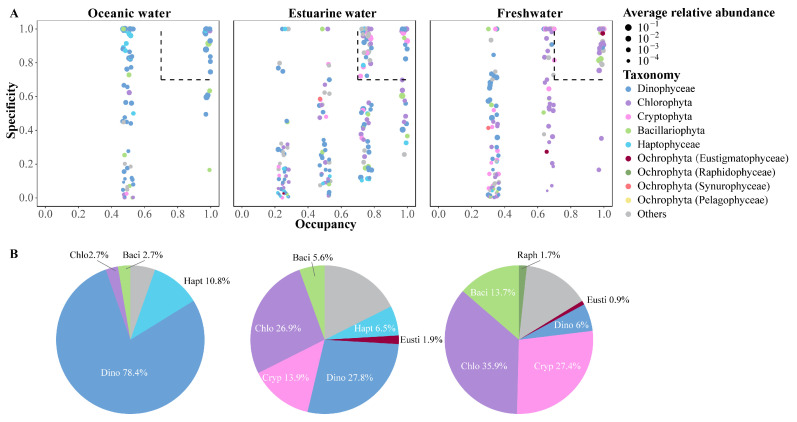
(**A**) SPEC–OCCU plots presenting the distribution of the top 500 ASVs across different salinity habitats in the Pearl River Estuary. Occupancy (x-axis) indicates the distribution of an ASV across all samples in salinity habitats, while specificity (y-axis) indicates whether it also present in other habitats. The dotted line includes phytoplankton with occupancy and specificity ≥0.7. Scatter colors and sizes represent different phylum (class) and relative abundances, respectively. (**B**) Pie charts depict the percentage of specialist ASVs in each salinity habitat.

The SPEC–OCCU plots illustrate the distribution and relative abundance of the top 500 most abundant ASVs across various habitats in the PRE, encompassing taxa such as Dinophyta, Chlorophyta, Cryptophyta, Bacillariophyta, Haptophyta, and other Ochrophyta (Figure 7). ASVs in each habitat showed a degree of variation in the occupancy (x-axis), with fewer species found in all stations (Figure 7A). Specialists, defined by both specificity and occupancy rates of ≥0.7, were identified in each habitat (Figure 7B). The number of specialist ASVs varied significantly across the salinity gradient, with 37 in oceanic areas, 108 in estuarine areas, and 117 in freshwater areas, representing diverse taxonomic groups. ASVs representing Dinophyta, Bacillariophyta, and Chlorophyta were present as specialists in all salinity habitats, though their proportions varied (Figure 7B). Among specialists, Dinophyta tended to dominate in higher salinity environments and were less prevalent in low salinity areas. Haptophyta accounted for 10.8% of the specialists in oceanic areas and 6.5% in estuarine areas but were not specialists in freshwater areas. In estuarine and freshwater areas, Chlorophyta and Cryptophyta constituted the majority of specialists, but their specificity was low in oceanic areas.

### 3.6. Correlations between Phytoplankton Community, Photosynthetic Physiology, and Environmental Factors

**Figure 8:**
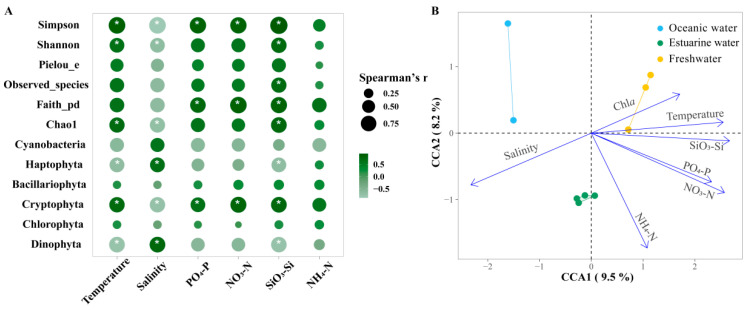
(**A**) Spearman correlation analysis between environmental factors and the relative abundance of major phytoplankton taxa and alpha biodiversity in the Pearl River Estuary. Cyanobacteria richness was determined based on pigment analysis, while the richness of other algae was determined based on 18S rRNA. Bubble size indicates the absolute value of the Spearman rank correlation coefficient. “*” denotes statistically significant differences at *p* < 0.05. (**B**) Canonical correlation analysis (CCA) of environmental factors and phytoplankton communities.

Figure 8A shows the Spearman correlations between the major phytoplankton groups, diversity indices, and environmental factors in the PRE. Among the main groups, Bacillariophyta and Chlorophyta showed no significant correlations with environmental factors (*p* > 0.05). Cryptophyta was significantly positively correlated with all nutrients except NH_4_^+^-N but was negatively correlated with salinity (*p* < 0.05). Dinophyta was significantly positively correlated with salinity but was negatively correlated with SiO_3_^2−^-Si and temperature (*p* <0.05). Diversity indices were significantly influenced by temperature and salinity as well as nutrient levels. Overall, the diversity indices were positively correlated with nutrient levels and temperature, but negatively correlated with salinity.

Furthermore, CCA was used to explore the overall regulation of environmental factors (excluding photosynthetic activity) on the community structure of phytoplankton in the PRE (Figure 8B; Table S2), which is consistent with NMDS and envfit. Compared to NMDS with envfit, CCA provides the explanation rate of the variation in the community structure. The eigenvalues of axes CCA 1 and CCA 2 together explained 17.7% of the variation in the phytoplankton community structure. According to permutation test results, environmental factors significantly influenced the variation in phytoplankton community structure (*p* < 0.05). Temperature, salinity, NO_3_^−^-N, PO_4_^3−^-P, and SiO_3_^2−^-Si had significant impacts on the phytoplankton community structure (*R*^2^ = 0.7381~0.9261, *p* < 0.05). Phytoplankton in oceanic areas were positively correlated with salinity but negatively correlated with temperature, NO_3_^−^-N, PO_4_^3−^-P, and SiO_3_^2−^-Si. The correlations for the phytoplankton in freshwater were the exact opposite. Nutrient levels significantly influenced phytoplankton in the upper estuary with high Chl *a* content.

**Figure 9:**
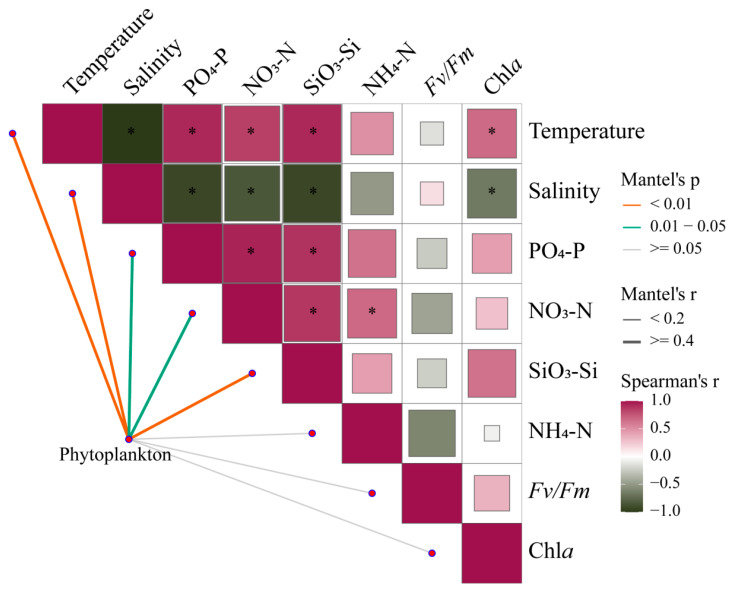
Pairwise comparisons between 18S rRNA-based phytoplankton communities and environmental factors in the Pearl River Estuary by Mantel tests. The upper triangle is the Spearman correlation between the environmental factor, Chl *a,* and *Fv*/*Fm*. The “*” denotes statistically significant differences at *p* < 0.05, and the color represents Spearman’s r. The line width represents Mantel’s r between each factor and the phytoplankton community, and the color represents Mantel’s *p-value*.

Mantel tests provide correlations of phytoplankton communities across the estuary with environmental changes and photosynthetic responses of *Fv*/*Fm* (Figure 9, Table S3). Under broad gradients in estuaries, phytoplankton communities are strongly shaped by temperature, salinity, and nutrient levels (*p* < 0.05). Meanwhile, there is a certain correlation between environmental factors, which will directly or indirectly affect the changes in phytoplankton communities.

## 4. Discussion

### 4.1. Community Composition of Phytoplankton in the Pearl River Estuary during Autumn

Combining molecular biology and chemotaxonomy provides detailed insights into the plankton community composition [39,56,57]. The PRE is an ideal experimental area, presenting the typical spatial and temporal differentiation of phytoplankton, and facilitating comparisons between different methods [39]. Both approaches in this study indicate that Dinophyta, Chlorophyta, Cryptophyta, Bacillariophyta, and Haptophyta are the dominant phytoplankton phyla in the estuary, consistent with previous research [26,39,58,59]. The taxonomy of phytoplankton at the genus level is also similar to Ding, et al. [58]. According to 18S rRNA sequencing results, in late summer and early fall, Dinophyta appeared at all stations, particularly dominating in the high and medium salinity areas (Figure 4). Similar observations of increased Dinophyta abundance during autumn, accompanied by massive blooms of *Cochlodinium geminatum*, were reported by Dong, et al. [33] and Ke, et al. [60]. Guo, et al. [61] also observed dinoflagellate blooms in the PRE in summer. Higher temperatures and salinity during this season were one of the main reasons for the enrichment of dinoflagellate, which is consistent with previous observations [33,60]. Additionally, both methods detected significant presences of Bacillariophyta at all stations (Figure 4 and Figure 5), which are important freshwater and brackish species in the estuary [36]. Due to the suitable water temperature and turbidity, diatoms prevail in both wet and dry seasons in the PRE [26]. The hydrological dynamics of the estuary, strongly influenced by seasonal variations, drive temporal shifts in phytoplankton composition [7,8,11,26,36].

Generally, the abundance of 18S rRNA genes was well consistent with pigment content in phytoplankton [39,56,62], representing the major algae along the PRE (Figure 4B and Figure 5B). Relative to CHEMTAX results, the 18S rRNA results showed a higher average relative abundance of Dinophyta, while Haptophyta appeared to be underestimated (Figure 4 and Figure 5), similar to observations from a 2020 cruise in the PRE by Xu, et al. [39]. The relative abundance of Chlorophyta was also higher in the pigment analysis. The underestimation of Haptophyta in the 18S dataset could be due to a single base mismatch on the 3′ end of the V4R primer in Prymnesiales (Haptophyta), leading to possible underestimation [59]. Developing primers with better specificity could help avoid the widespread underestimation of this significant marine group [63]. Furthermore, the variability in 18S rRNA gene copies among different eukaryotic species can lead to discrepancies between PCR-based and pigment-based methods [64,65], which could also explain the discrepancy in the content of other algae in the two methods in this study. Compared to other algae, Dinophyta, possessing considerably more rRNA gene copies, is more readily detected in sequencing studies [64]. Dinophyta’s unique nutritional strategies might also contribute to such inconsistencies. Most dinophytes are not strictly autotrophic and can achieve mixotrophy through predatory strategies or even incorporating phototrophy through the acquisition of chloroplasts from other algae [66,67]. This phenomenon can result in the potential underreporting of mixotrophic Dinophyta in chemotaxonomic analyses based on pigment contents. In summary, combining multiple approaches helps to comprehensively understand the composition of marine phytoplankton.

### 4.2. Spatial Heterogeneity of Photosynthetic Characteristics in Phytoplankton of the Pearl River Estuary

*Fv*/*Fm*, recognized as a sensitive in situ indicator of stressors for phytoplankton [16,17,19], effectively reflects the status of photosynthetic physiology of phytoplankton along the environmental gradient of the PRE (Figure 3 and Figure 6A). Li, et al. [19] categorized estuarine phytoplankton based on *Fv*/*Fm* values into stress (low, <0.3), transitional (moderate, 0.3–0.5), and blooming conditions (high, >0.5). In this study, phytoplankton in the upper estuary (dominant by Dinophyta, Bacillariophyta, and Haptophyta) and lower estuary with high nutrient levels (dominant by Cryptophyta, Chlorophyta, and Bacillariophyta) appeared healthier and more active, whereas communities in the midstream were more stressed (Figure 3 and Figure 6A).

The salinity gradient of estuaries is well-known as one of the most significant and fundamental causes of phytoplankton stress, resulting in slowed growth, reduced photosynthesis, and increased respiration. These factors contribute to the spatial succession of estuarine phytoplankton communities [68,69]. Our results demonstrate a complex correlation between photosynthetic activity in phytoplankton of the PRE and variations in salinity (Figure 3). Different species exhibit varying adaptations to salinity [68,69]. After river water flows into the upper Lingdingyang, osmotic stress caused by the shift inhibits stenohaline species originating from freshwater environments [68], leading to decreased algal photosynthetic activity [10]. As the environment transitions to higher salinity marine conditions, the community becomes dominated by euryhaline species such as Dinophyta and Bacillariophyta [70].

Light availability is always a primary factor limiting the photosynthetic physiology of phytoplankton. Within brackish water environments, in contrast to freshwater and seawater, phytoplankton cells are often more sensitive to changes in ultraviolet (UV) radiation, resulting in significant inhibition of photosynthesis [10]. Reduced light availability due to high turbidity in estuaries also contributes to the limitation of phytoplankton physiology and growth [19,26], despite higher nutrient levels in the middle sections of the estuary (Figure 2). The Estuarine Turbidity Maxima (ETM) zone, observed in many estuaries worldwide [71], exerts a substantial influence on the distribution of marine microbes [72,73]. Shi, et al. [27] found that the ETM zone in the PRE is located on the western side of the middle channel of the PRE during the dry season, with high bacterial abundance but low phytoplankton abundance. Therefore, the decline in photosynthetic activity within the mid-channel (an area characterized by moderate salinity) of the PRE may be caused by the cumulative influence of turbidity-induced alterations in light availability and dramatic changes in salinity. In contrast, nutrient levels were also higher upstream, but lower turbidity resulted in higher *Fv*/*Fm* in the phytoplankton community.

### 4.3. Environmental Factors Shaping Spatial Distribution of Phytoplankton

The significant environmental gradient in the PRE (Figure 2) leads to heterogeneity within the phytoplankton community, thereby instigating shifts in both structure and abundance (Figure 4, Figure 5, Figure 6 and Figure 7). Salinity strongly governs the spatial succession of the phytoplankton community in the estuary [7,11,58,74], dividing it into three distinct groups: the estuarine community, the mixing waters community, and the coastal community [36]. SPEC–OCCU plots provide detailed insights into species specialization (Figure 7). Along the salinity gradient, unique species primarily belonging to Chlorophyta and Cryptophyta, adapted to oligohaline and mesohaline conditions, transition into species like Dinophyta and Haptophyta, which thrive in polyhaline and euhaline conditions. Meanwhile, Bacillariophyta species dominate across all salinity habitats (Figure 4 and Figure 5). This succession pattern is similar to that observed in other estuaries [10,70]. Generally, freshwater species are confined to the upper reaches of the river, with only a few extending into saline areas, whereas marine species can extend into oligohaline and even freshwater regions, although with reduced diversity [6].

Nutrient levels regulate the composition, abundance, diversity, and primary productivity of estuarine phytoplankton [8,19,34,75]. Due to strong terrestrial influences, the upper and middle estuaries are nutrient-rich, leading to significant increases in Chl *a* (Figure 5). As the river widens downstream, nutrients are quickly diluted, resulting in decreased Chl *a* content. CCA analysis and NMDS with envfit indicate that the low-salinity community is positively correlated with nutrient concentrations (Figure 8B), highlighting the stimulatory effect of nutrients. Elevated nutrient levels also significantly enhance diversity (Figure 6A and Figure 8A). Studies have shown that SiO_3_^2−^-Si concentration significantly affects the growth of phytoplankton in the PRE [11,36], and this study likewise confirms the positive role of silicate on the major phytoplankton groups and diversity (Figure 8A). Additionally, the enrichment of phytoplankton in the estuary is controlled by residence time/turnover rate, closely related to seasonality and flow rates [76]. Compared to the Mississippi River and Yangtze River, the residence time of PRE is relatively short, ranging from a couple of weeks in the dry season to a few days in the wet season [77,78], resulting in complex temporal and spatial dynamics of phytoplankton. Higher nutrient levels and the stability of the water column during autumn contribute to phytoplankton enrichment in the middle to upper parts of the estuary as well as higher photosynthetic activity.

With the fast development in the Pearl River Delta area, research on a 40-year PRE data set suggested that there was a significant decrease in the number of phytoplankton species [79], accompanied by an increase in harmful algal bloom events [80]. Although *Chaetoceros* sp. is still one of the dominant diatoms, *Cyclotella* adapted and bloomed in the estuary prompted by the anthropogenic dissolved organic phosphorus (DOP) [81]. The low light availability and high nutrients have favored the blooms of pico-level brackish Chlorophyta (e.g., *Ostreococcus tauri*) [58], while the continuous supply of DIN with an enrichment of DOP supported the recurrent harmful algal blooms of *Gyrodinium* in the lower estuary [82]. In the upper river, mixotrophic *Chlorella* and *Chlamydomonas* also bloom [83,84]. All of these species are present in our sample at the genus level. The ongoing anthropogenic influences on the PRE have shaped the dynamics of the phytoplankton community [34,75,85], and assessing the function and health of coastal ecosystems aids in developing long-term management strategies.

## 5. Conclusions

The 18S rRNA and pigment analysis showed that environmental gradients in the PRE play a dominant role in shaping the differentiation of phytoplankton communities into oceanic, estuarine, and freshwater communities. The relative abundance of Dinophyta and Haptophyta decreased from the coast to the upper estuary, while Chlorophyta and Cryptophyta became increasingly dominant. Bacillariophyta was identified as an important species present in both saline and freshwater environments. The in situ photosynthetic characteristic of phytoplankton along the PRE indicated complex correlations with environmental factors. Due to a wide variety of environmental gradients and the adaptability of phytoplankton, communities in the mixed zone were observed to be under obvious stress, while those in freshwater and oceanic regions appeared relatively active and healthy. The combination of environmental factors revealed that, in addition to the temperature and salinity gradients of the PRE, the increase in nutrient levels related to anthropogenic influence also had significant effects on the concentration of Chl *a* and the phytoplankton community in the upper estuary. Focusing on the photosynthetic characteristics and community structure of coastal phytoplankton contributes to understanding the functioning and health of coastal ecosystems.

## Figures and Tables

**Table 1:** Alpha diversity indices of phytoplankton based on 18S rRNA in the Pearl River Estuary.

Group	Chao1	Faith’s PD	Observed Species	Pielou’s Evenness	Shannon	Simpson
Oceanic water	670.766 ± 242.410	66.662 ± 40.004	661.150 ± 229.598	0.717 ± 0.0252	6.690 ± 0.602	0.956 ± 0.005
Estuarine water	703.047 ± 170.311	104.138 ±32.528	644.200 ± 133.957	0.6733 ± 0.065	6.280 ± 0.803	0.945 ± 0.045
Freshwater	1129.983 ± 87.660	122.425 ± 15.439	987.100 ± 87.167	0.757 ± 0.035	7.530 ± 0.426	0.977 ± 0.008

## Data Availability

Data will be made available on request. The raw sequencing data were uploaded to NCBI (https://www.ncbi.nlm.nih.gov/, accessed on 20 April 2024), with the accession number PRJNA1101256.

## Supplementary Materials

The following supporting information can be downloaded at: https://www.mdpi.com/article/10.3390/biology13070550/s1, Figure S1: Rarefaction curve of phytoplankton sequencing; Table S1: Top 20 phytoplankton taxonomy at the genus level in the Pearl River Estuary based on 18S rDNA sequencing; Table S2: Permutation test results of environmental factors for the CCA; Table S3: Pairwise comparisons between 18S rRNA-based phytoplankton communities and environmental factors in the Pearl River Estuary by Mantel tests.

## References

[1] Cloern J.E., Jassby A.D., Schraga T.S., Nejad E., Martin C. (2017). Ecosystem variability along the estuarine salinity gradient: Examples from long-term study of San Francisco Bay. Limnol. Oceanogr..

[2] Elliott M., Whitfield A.K. (2011). Challenging paradigms in estuarine ecology and management. Estuar. Coast. Shelf Sci..

[3] Muylaert K., Sabbe K., Vyverman W. (2009). Changes in phytoplankton diversity and community composition along the salinity gradient of the Schelde estuary (Belgium/The Netherlands). Estuar. Coast. Shelf Sci..

[4] Quinlan E.L., Phlips E.J. (2007). Phytoplankton assemblages across the marine to low-salinity transition zone in a blackwater dominated estuary. J. Plankton Res..

[5] Herlemann D.P.R., Labrenz M., Jürgens K., Bertilsson S., Waniek J.J., Andersson A.F. (2011). Transitions in bacterial communities along the 2000 km salinity gradient of the Baltic Sea. ISME J..

[6] Whitfield A.K., Elliott M., Basset A., Blaber S.J.M., West R.J. (2012). Paradigms in estuarine ecology—A review of the Remane diagram with a suggested revised model for estuaries. Estuar. Coast. Shelf Sci..

[7] Zhang X., Zhang J.P., Huang X.P., Huang L.M. (2014). Phytoplankton assemblage structure shaped by key environmental variables in the Pearl River Estuary, South China. J. Ocean Univ..

[8] Li G., Lin Q., Lin J., Song X., Tan Y., Huang L. (2014). Environmental gradients regulate the spatial variations of phytoplankton biomass and community structure in surface water of the Pearl River estuary. Acta Ecol. Sin..

[9] Wang H.L., Chen F., Zhang C.L., Wang M., Kan J.J. (2021). Estuarine gradients dictate spatiotemporal variations of microbiome networks in the Chesapeake Bay. Environ. Microbiome.

[10] Li G., Gao K.S., Yuan D.X., Zheng Y., Yang G.Y. (2011). Relationship of photosynthetic carbon fixation with environmental changes in the Jiulong River estuary of the South China Sea, with special reference to the effects of solar UV radiation. Mar. Pollut. Bull..

[11] Xu S.N., Liu Y., Fan J.T., Xiao Y.Y., Qi Z.H., Lakshmikandan M. (2022). Impact of salinity variation and silicate distribution on phytoplankton community composition in Pearl River estuary, China. Ecohydrol. Hydrobiol..

[12] Cloern J.E., Jassby A.D. (2010). Patterns and Scales of Phytoplankton Variability in Estuarine-Coastal Ecosystems. Estuaries Coasts.

[13] Anderson D.M., Glibert P.M., Burkholder J.M. (2002). Harmful algal blooms and eutrophication: Nutrient sources, composition, and consequences. Estuaries.

[14] Gilbert P.M. (2017). Eutrophication, harmful algae and biodiversity—Challenging paradigms in a world of complex nutrient changes. Mar. Pollut. Bull..

[15] Chen D.W., Shi Z., Li R.H., Li X.F., Cheng Y.Y., Xu J. (2023). Hydrodynamics drives shifts in phytoplankton community composition and carbon-to-chlorophyll *a* ratio in the northern South China Sea. Front. Mar. Sci..

[16] Li J.L., Sun X.X., Zheng S. (2016). In situ study on photosynthetic characteristics of phytoplankton in the Yellow Sea and East China Sea in summer 2013. J. Mar. Syst..

[17] Wang F., Guo S.J., Liang J.H., Sun X.X. (2024). In situ phytoplankton photosynthetic characteristics and their controlling factors in the eastern Indian Ocean. Mar. Pollut. Bull..

[18] Cloern J.E. (1999). The relative importance of light and nutrient limitation of phytoplankton growth: A simple index of coastal ecosystem sensitivity to nutrient enrichment. Aquat. Ecol..

[19] Li J., Gao Y.H., Bao Y.L., Gao X., Glibert P.M. (2023). Summer phytoplankton photosynthetic characteristics in the Changjiang River Estuary and the adjacent East China Sea. Front. Mar. Sci..

[20] Zhang L.K., Li G., Xiang C.H., Huang Y.D., Fu X.M., Zheng C.Y., Wang Z., Ouyang Z.Y., Song X.Y. (2022). Plankton Metabolism in Coastal Waters of the Guangdong-Hong Kong-Macao Greater Bay: Regional Variance and Driving Factors. Front. Mar. Sci..

[21] Bergmann T., Richardson T.L., Paerl H.W., Pinckney J.L., Schofield O. (2002). Synergy of light and nutrients on the photosynthetic efficiency of phytoplankton populations from the Neuse River Estuary, North Carolina. J. Plankton Res..

[22] Xia J., Bao Y.L., Gao Y.H., Li J. (2024). The effects of temperature and sulfamethoxazole on the growth and photosynthetic characteristics of *Phaeodactylum tricornutum*. Mar. Pollut. Bull..

[23] Hanelt D., Häder D.-P., Erzinger G.S. (2018). 9-Photosynthesis assessed by chlorophyll fluorescence. Bioassays.

[24] Falkowski P.G., Kolber Z. (1995). Variations in chlorophyll fluorescence yields in phytoplankton in the world oceans. Aust. J. Plant Physiol..

[25] Bao Y., Li J. (2023). Photosynthetic Characteristics of Phytoplankton in the Surface Water of Changjiang Estuary and Its Adjacent Sea Area in Summer. Adv. Mar. Sci..

[26] Shen P.P., Li G., Huang L.M., Zhang J.L., Tan Y.H. (2011). Spatio-temporal variability of phytoplankton assemblages in the Pearl River estuary, with special reference to the influence of turbidity and temperature. Cont. Shelf Res..

[27] Shi Z., Xu J., Huang X.P., Zhang X., Jiang Z.J., Ye F., Liang X.M. (2017). Relationship between nutrients and plankton biomass in the turbidity maximum zone of the Pearl River Estuary. J. Environ. Sci..

[28] Cheshmehzangi A., Tang T. (2022). Pearl River Delta City Cluster: From Dual-Core Structure Economic Development Strategies to Regional Economic Plans. China’s City Cluster Development in the Race to Carbon Neutrality.

[29] Chen S.L., Zhu Z.H., Liu X.T., Yang L. (2022). Variation in Vegetation and Its Driving Force in the Pearl River Delta Region of China. Int. J. Environ. Res. Public Health.

[30] Shen Y.J. (2023). Analysis of Urban Expansion in the Pearl River Delta Urban Agglomeration Based on Multi-Source Time-Series Remote Sensing Data. Master’s Thesis.

[31] Tao W., Niu L.X., Dong Y.H., Fu T., Lou Q.S. (2021). Nutrient Pollution and Its Dynamic Source-Sink Pattern in the Pearl River Estuary (South China). Front. Mar. Sci..

[32] Zhang S.Y., Zhang H. (2023). Anthropogenic impact on long-term riverine COD_Mn_, BOD, and nutrient flux variation in the Pearl River Delta. Sci. Total Environ..

[33] Dong Y.L., Cui L., Cao R.B., Cen J.Y., Zou J., Zhou X.Y., Lu S.H. (2020). Ecological characteristics and teratogenic retinal determination of *Cochlodinium geminatum* blooms in Pearl River Estuary, South China. Ecotox. Environ. Safe..

[34] Harrison P.J., Yin K.D., Lee J.H.W., Gan J.P., Liu H.B. (2008). Physical-biological coupling in the Pearl River Estuary. Cont. Shelf Res..

[35] Hu J.T., Li S.Y., Geng B.X. (2011). Modeling the mass flux budgets of water and suspended sediments for the river network and estuary in the Pearl River Delta, China. J. Mar. Syst..

[36] Huang L.M., Jian W.J., Song X.Y., Huang X.P., Liu S., Qian P.Y., Yin K.D., Wu M. (2004). Species diversity and distribution for phytoplankton of the Pearl River estuary during rainy and dry seasons. Mar. Pollut. Bull..

[37] Charvet S., Vincent W.F., Lovejoy C. (2012). Chrysophytes and other protists in High Arctic lakes: Molecular gene surveys, pigment signatures and microscopy. Polar Biol..

[38] Xiao X., Sogge H., Lagesen K., Tooming-Klunderud A., Jakobsen K.S., Rohrlack T. (2014). Use of high throughput sequencing and light microscopy show contrasting results in a study of phytoplankton occurrence in a freshwater environment. PLoS ONE.

[39] Xu S.M., Li G.H., He C., Huang Y., Yu D., Deng H.W., Tong Z.Y., Wang Y.C., Dupuy C., Huang B.Q. (2023). Diversity, community structure, and quantity of eukaryotic phytoplankton revealed using 18S rRNA and plastid 16S rRNA genes and pigment markers: A case study of the Pearl River Estuary. Mar. Life Sci. Technol..

[40] Yang J., Lv J.P., Liu Q., Nan F.R., Li B., Xie S.L., Feng J. (2021). Seasonal and spatial patterns of eukaryotic phytoplankton communities in an urban river based on marker gene. Sci. Rep..

[41] Mackey D.J., Higgins H.W., Mackey M.D., Holdsworth D. (1998). Algal class abundances in the western equatorial Pacific: Estimation from HPLC measurements of chloroplast pigments using CHEMTAX. Deep-Sea Res. Part I-Oceanogr. Res. Pap..

[42] Swan C.M., Vogt M., Gruber N., Laufkoetter C. (2016). A global seasonal surface ocean climatology of phytoplankton types based on CHEMTAX analysis of HPLC pigments. Deep-Sea Res. Part I-Oceanogr. Res. Pap..

[43] Wright S.W., van den Enden R.L., Pearce I., Davidson A.T., Scott F.J., Westwood K.J. (2010). Phytoplankton community structure and stocks in the Southern Ocean (30–80°E) determined by CHEMTAX analysis of HPLC pigment signatures. Deep-Sea Res. Part II-Top. Stud. Oceanogr..

[44] (2007). The Specification for Marine Monitoring-Part 4: Seawater Analysis.

[45] Van Heukelem L., Thomas C.S. (2001). Computer-assisted high-performance liquid chromatography method development with applications to the isolation and analysis of phytoplankton pigments. J. Chromatogr. A.

[46] Butler W.L., Kitajima M. (1975). Fluorescence quenching in photosystem-II of chloroplasts. Biochim. Biophys. Acta.

[47] Stoeck T., Bass D., Nebel M., Christen R., Jones M.D.M., Breiner H.W., Richards T.A. (2010). Multiple marker parallel tag environmental DNA sequencing reveals a highly complex eukaryotic community in marine anoxic water. Mol. Ecol..

[48] Bolyen E., Rideout J.R., Dillon M.R., Bokulich N., Abnet C.C., Al-Ghalith G.A., Alexander H., Alm E.J., Arumugam M., Asnicar F. (2019). Reproducible, interactive, scalable and extensible microbiome data science using QIIME 2. Nat. Biotechnol..

[49] Martin M. (2011). Cutadapt removes adapter sequences from high-throughput sequencing reads. EMBnet. J..

[50] Callahan B.J., McMurdie P.J., Rosen M.J., Han A.W., Johnson A.J.A., Holmes S.P. (2016). DADA2: High-resolution sample inference from Illumina amplicon data. Nat. Methods.

[51] Bokulich N.A., Kaehler B.D., Rideout J.R., Dillon M., Bolyen E., Knight R., Huttley G.A., Caporaso J.G. (2018). Optimizing taxonomic classification of marker-gene amplicon sequences with QIIME 2′s q2-feature-classifier plugin. Microbiome.

[52] Schlitzer R. Ocean Data View. https://odv.awi.de/.

[53] Gu Z.G., Gu L., Eils R., Schlesner M., Brors B. (2014). *circlize* implements and enhances circular visualization in R. Bioinformatics.

[54] Gweon H.S., Bowes M.J., Moorhouse H.L., Oliver A.E., Bailey M.J., Acreman M.C., Read D.S. (2021). Contrasting community assembly processes structure lotic bacteria metacommunities along the river continuum. Environ. Microbiol..

[55] Mackey M.D., Mackey D.J., Higgins H.W., Wright S.W. (1996). CHEMTAX—A program for estimating class abundances from chemical markers: Application to HPLC measurements of phytoplankton. Mar. Ecol.-Prog. Ser..

[56] Lin Y.J., Gifford S., Ducklow H., Schofield O., Cassar N. (2019). Towards quantitative microbiome community profiling using internal standards. Appl. Environ. Microbiol..

[57] Piquet A.M.T., van de Poll W.H., Visser R.J.W., Wiencke C., Bolhuis H., Buma A.G.J. (2014). Springtime phytoplankton dynamics in Arctic Krossfjorden and Kongsfjorden (Spitsbergen) as a function of glacier proximity. Biogeosciences.

[58] Ding X., Liu J.X., Liu W.W., Dai S., Ke Z.X., Guo J., Lai Y.J., Tan Y.H. (2023). Phytoplankton Communities Miniaturization Driven by Extreme Weather in Subtropical Estuary under Climate Changes. Water Res..

[59] Yeh Y.C., McNichol J., Needham D.M., Fichot E.B., Berdjeb L., Fuhrman J.A. (2021). Comprehensive single-PCR 16S and 18S rRNA community analysis validated with mock communities, and estimation of sequencing bias against 18S. Environ. Microbiol..

[60] Ke Z.X., Huang L.M., Tan Y.H., Song X.Y. (2012). A dinoflagellate *Cochlodinium geminatum* bloom in the Zhujiang (Pearl) River estuary in autumn 2009. Chin. J. Oceanol. Limnol..

[61] Guo Y.P., Lin S.J., Huang L.M., Chen Y.Q., Hu S.M., Liu S., Tan Y.H., Huang X.P., Qiu D.J. (2022). Dissipation of a *Polykrikos geminatum* Bloom after Wind Events in Pearl River Estuary. Water.

[62] Gong W.D., Hall N., Paerl H., Marchetti A. (2020). Phytoplankton composition in a eutrophic estuary: Comparison of multiple taxonomic approaches and influence of environmental factors. Environ. Microbiol..

[63] Liu H., Probert I., Uitz J., Claustre H., Aris-Brosou S., Frada M., Not F., de Vargas C. (2009). Extreme diversity in noncalcifying haptophytes explains a major pigment paradox in open oceans. Proc. Natl. Acad. Sci. USA.

[64] Potvin M., Lovejoy C. (2009). PCR-based diversity estimates of artificial and environmental 18s rRNA gene libraries. J. Eukaryot. Microbiol..

[65] Zhu F., Massana R., Not F., Marie D., Vaulot D. (2005). Mapping of picoeucaryotes in marine ecosystems with quantitative PCR of the 18S rRNA gene. FEMS Microbiol. Ecol..

[66] Hansen P.J., Nielsen L.T., Johnson M., Berge T., Flynn K.J. (2013). Acquired phototrophy in *Mesodinium* and *Dinophysis*—A review of cellular organization, prey selectivity, nutrient uptake and bioenergetics. Harmful Algae.

[67] Mitra A., Flynn K.J., Tillmann U., Raven J.A., Caron D., Stoecker D.K., Not F., Hansen P.J., Hallegraeff G., Sanders R. (2016). Defining planktonic protist functional groups on mechanisms for energy and nutrient acquisition: Incorporation of diverse mixotrophic strategies. Protist.

[68] D’Ors A., Bartolomé M.C., Sánchez-Fortún S. (2016). Repercussions of salinity changes and osmotic stress in marine phytoplankton species. Estuar. Coast. Shelf Sci..

[69] Li G. (2019). Fast acclimation of phytoplankton assemblies to acute salinity stress in the Jiulong River Estuary. Acta Oceanol. Sin..

[70] Nche-Fambo F.A., Scharler U.M., Tirok K. (2015). Resilience of estuarine phytoplankton and their temporal variability along salinity gradients during drought and hypersalinity. Estuar. Coast. Shelf Sci..

[71] Burchard H., Schuttelaars H.M., Ralston D.K. (2018). Sediment Trapping in Estuaries. Annu. Rev. Mar. Sci..

[72] Goosen N.K., Kromkamp J., Peene J., van Rijswik P., van Breugel P. (1999). Bacterial and phytoplankton production in the maximum turbidity zone of three European estuaries: The Elbe, Westerschelde and Gironde. J. Mar. Syst..

[73] Huang X., Huang L. (2002). Progress in researches on dynamical processes of phytoplankton ecology in maximum turbidity zone of estuary. Acta Ecol. Sin..

[74] Wu J.N., Zhu Z., Waniek J.J., Niu M.Y., Wang Y.T., Zhang Z.R., Zhou M., Zhang R.F. (2023). The biogeography and co-occurrence network patterns of bacteria and microeukaryotes in the estuarine and coastal waters. Mar. Environ. Res..

[75] Niu L.X., Luo X.X., Hu S., Liu F., Cai H.Y., Ren L., Ou S.Y., Zeng D.N., Yang Q.S. (2020). Check impact of anthropogenic forcing on the environmental controls of phytoplankton dynamics between 1974 and 2017 in the Pearl River estuary, China. Ecol. Indic..

[76] Lu Z.M., Gan J.P. (2015). Controls of seasonal variability of phytoplankton blooms in the Pearl River Estuary. Deep-Sea Res. Part II-Top. Stud. Oceanogr..

[77] Rabouille C., Conley D.J., Dai M.H., Cai W.J., Chen C.T.A., Lansard B., Green R., Yin K., Harrison P.J., Dagg M. (2008). Comparison of hypoxia among four river-dominated ocean margins: The Changjiang (Yangtze), Mississippi, Pearl, and Rhone rivers. Cont. Shelf Res..

[78] Sun J., Lin B.L., Li K.M., Jiang G.Q. (2014). A modelling study of residence time and exposure time in the Pearl River Estuary, China. J. Hydro-Environ. Res..

[79] Chen W.L., Guo F., Huang W.J., Wang J.G., Zhang M., Wu Q. (2023). Advances in phytoplankton population ecology in the Pearl river estuary. Front. Environ. Sci..

[80] Gu H., Wu Y., Lu S., Lu D., Tang Y.Z., Qi Y. (2022). Emerging harmful algal bloom species over the last four decades in China. Harmful Algae.

[81] Li K., Huang L., Zhang J., Yin J., Luo L. (2010). Characteristics of phytoplankton community in the Pearl River Estuary during saline water intrusion period. J. Trop. Oceanogr..

[82] Wang Z.H., Guo X., Qu L.J., Lin L.C. (2017). Effects of nitrogen and phosphorus on the growth of *Levanderina fissa*: How it blooms in Pearl River Estuary. J. Ocean Univ..

[83] Ren H., Tian T., Yang Y., Wang Q. (2017). Spatial and temporal distribution of phytoplankton community and its relationship with environment factors in Nansha’s Rivers, Pearl River estuary. Acta Ecol. Sin..

[84] Gou T., Xu Z., Li J., Ma Q., Wang L., Zhao X., Liang R., Guo J. (2015). Phytoplankton community structure and water quality assessment of Hejiang River, a branch of Xijiang River, Pearl River drainage basin. Hupo Kexue.

[85] Qiu D.J., Huang L.M., Zhang J.L., Lin S.J. (2010). Phytoplankton dynamics in and near the highly eutrophic Pearl River Estuary, South China Sea. Cont. Shelf Res..

