# Supplementary material for: Phytoplankton Diversity, Spatial Patterns, and Photosynthetic Characteristics Under Environmental Gradients and Anthropogenic Influence in the Pearl River Estuary
Source: Biology (Basel). 2024 Jul 22;13(7):550. doi: 10.3390/biology13070550 (PMC11273628; doi:10.3390/biology13070550)
Supplement: Supplementary file 1 [file biology-13-00550-s001.zip › biology-3078108-supplementary.pdf]

Phytoplankton Diversity, Spatial Patterns, and Photosynthetic Characteristics Under Environmental Gradients and Anthropogenic Influence in the Pearl River Estuary

Jing Xia [1,†], Haojie Hu [1,†], Xiu Gao [1], Jinjun Kan [2], Yonghui Gao [3,4,*] and Ji Li [3,4,*]

[1] School of Oceanography, Shanghai Jiao Tong University, Shanghai 200030, China
[2] Stroud Water Research Center, 970 Spencer Rd., Avondale, PA 19311, USA
[3] Key Laboratory of Polar Ecosystem and Climate Change, Ministry of Education; and School of Oceanography, Shanghai Jiao Tong University, Shanghai 200030, China
[4] Key Laboratory for Polar Science, Polar Research Institute of China, Ministry of Natural Resources, Shanghai 200136, China
* Correspondence: ygao80@sjtu.edu.cn (Y.G.); liji81@sjtu.edu.cn (J.L.)
† These authors contributed equally to this work.

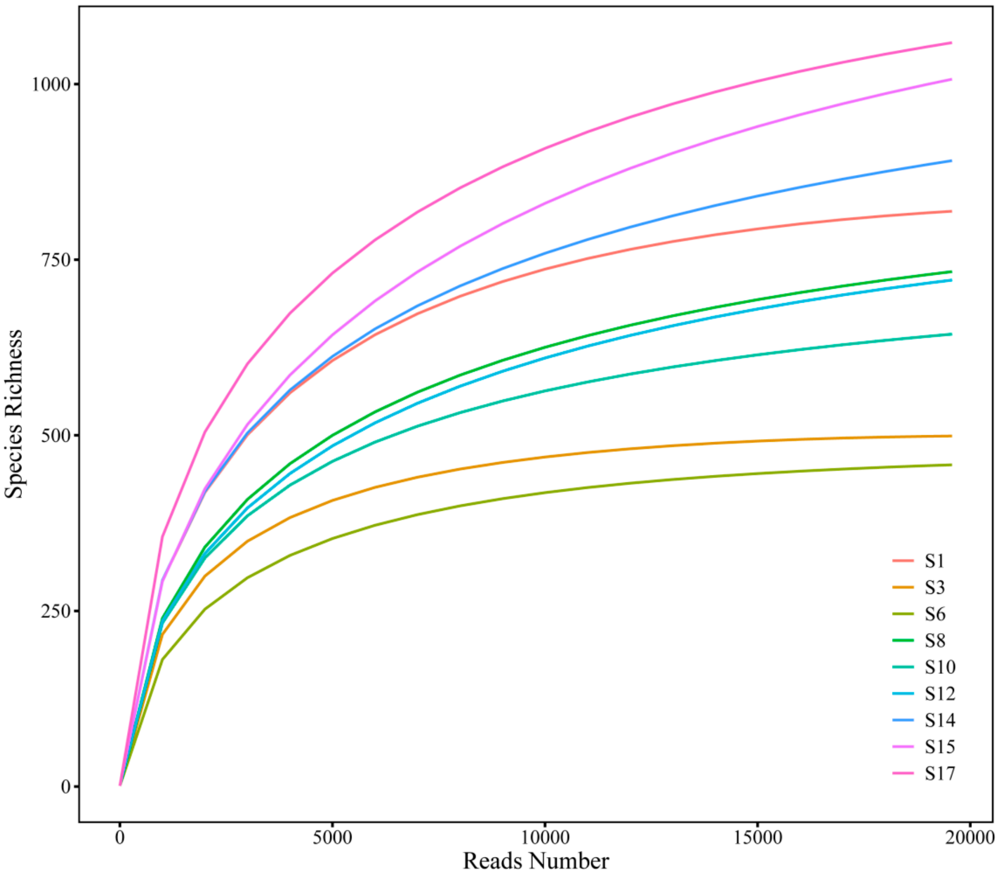

Figure S1 Rarefaction curve of phytoplankton sequencing.

Table S1 Most 20 phytoplankton taxonomy at the genus level in the Pearl River Estuary based on 18S rDNA sequencing.

Genus	Oceanic water	Estuarine water	Freshwater
Cryptomonas	0.01%	0.03%	25.81%
Gyrodinium	16.08%	0.01%	<0.01%
Cyclotella	0.02%	6.98%	8.96%
Chlorella	0.03%	11.84%	2.44%
Chaetoceros	10.59%	1.27%	0.02%
Ostreococcus	0.43%	6.63%	0.01%
Podolampas	<0.01%	2.65%	2.54%
Picochlorum	0.03%	4.41%	0.05%
Chrysochromulina	2.89%	0.86%	0.01%
Teleaulax	0.06%	2.38%	0.56%
Chlamydomonas	<0.01%	0.10%	2.76%
Skeletonema	0.26%	0.48%	1.65%
Mychonastes	<0.01%	0.60%	1.09%
Heterocapsa	<0.01%	0.99%	0.20%
Phaeocystis	0.84%	0.32%	<0.01%
Micromonas	0.38%	0.69%	<0.01%
Komma	<0.01%	<0.01%	1.04%
Warnowia	0.87%	0.03%	<0.01%

Aulacoseira	<0.01%	<0.01%	0.82%
Oodinium	0.03%	0.65%	<0.01%

Table S2 Permutation test results of environmental factors for the CCA.

**Environmental factors**	**CCA1**	**CCA2**	**R^2**	**p**
Temperature	0.99760	0.06931	0.8006	0.009
Salinity	-0.94098	-0.33847	0.7490	0.030
PO_4-P	0.94696	-0.32134	0.7381	0.023
NO_3-N	0.93680	-0.34987	0.9261	0.004
SiO_3-Si	0.99891	-0.04659	0.8772	0.007
NH_4-N	0.50299	-0.86429	0.5672	0.075
Chl *a*	0.93800	0.34664	0.4057	0.161

Table S3 Pairwise comparisons between 18S rRNA-based phytoplankton communities and environmental factors in the Pearl River Estuary by Mantel tests.

**Environmental factors**	**R**	**p**
Temperature	0.551958532	0.004
Salinity	0.642365447	0.001
PO_4-P	0.470480611	0.011
NO_3-N	0.400077288	0.013
SiO_3-Si	0.636859839	0.001
NH4-N	-0.045462144	0.605
Fv/Fm	-0.06532915	0.599
Chl *a*	0.152192105	0.272
